# Atypical Presentation of Viral Gastroenteritis in a Three-year-old Child Due to a UNC80 Mutation

**DOI:** 10.7759/cureus.4395

**Published:** 2019-04-05

**Authors:** Kristen K Stenehjem, Jessica Schweigert, Parag Kumar

**Affiliations:** 1 Pediatrics, University of North Dakota School of Medicine and Health Sciences, Bismarck, USA

**Keywords:** unc80, gastroenteritis, pneumonia, nalcn, hypotonia, developmental delay, viral gastroenteritis

## Abstract

We report a three-year-old child with a rare genetic phenomenon, a UNC80 mutation, who had an unusual presentation of viral gastroenteritis. The UNC80 gene encodes for an important voltage-independent channel in neurons and a mutation in this protein can lead to severe hypotonia. The hypotonia manifests as delayed gastric emptying, impaired respiratory clearance, and/or delayed muscle coordination, which can predispose to infection susceptibility. UNC80 gene mutations have also been shown to cause global developmental delays, failure to thrive, and phenotypic dysmorphisms. In our patient, we believe that his genetic defect precipitated a complicated hospital course. The patient’s delayed gastric emptying caused difficulty in recovering from viral gastroenteritis while a concurrent pneumonia diagnosis required assistance in clearing respiratory contents. The UNC80 mutation is under-studied, and more studies are necessary to understand the clinical implications of its phenotypes.

## Introduction

The UNC80 gene encodes proteins important in the expression of a trimeric channel protein permeable to Na^+^, K^+^, and Ca^2+^ (NALCN) expressed most often in neurons [1]. The NALCN channel complex is voltage-independent, allowing Na+ leakage at resting membrane potential. Proteins expressed from the UNC80 gene bridge UNC79 and NALCN proteins to create the large channel complex that plays an important role in neuron excitability [1-2]. Disruptions in any component of the channel complex can lead to major disruptions in a neuron’s ability to conduct electrical conductance, leading to severe pathological phenotypes [1].

UNC80 gene mutations have been found to cause severe hypotonia, global developmental delays, failure to thrive, and increased susceptibility to infection. Upper respiratory infections and aspiration pneumonia have been most commonly described in the literature [3]. Many patients with UNC80 mutations have dysmorphisms including plagiocephaly, broad forehead with a prominent nose, smooth philtrum, and congenital esotropia [3-5]. Herein, we report a three-year-old male patient with a UNC80 mutation, presenting with viral gastroenteritis and pneumonia with a complicated hospital course. 

## Case presentation

A three-year-old male presented to the emergency room (ER) with persistent thrusting of his pelvis anteriorly and inconsolable crying. The patient has a complex past medical history significant for hypotonia, developmental delays, and poor weight gain due to a UNC80 mutation of 44 Mb homozygosity of 2q33.1-q37.3 and smaller regions of homozygosity on chromosomes two, three, 11, and 16. Due to this mutation, the patient has a past history of reactive airway disease and recurrent pneumonia. He requires BiPap to assist his oxygenation during the night. He uses a cough assist machine four times daily to help clear his secretions. He is unable to clear the secretions due to his hypotonia caused by the chromosomal abnormality. Nutrition is received via his G-tube. He is non-verbal at baseline due to significant developmental delay. 

Eighteen days prior to admission, the patient developed a low-grade fever, non-productive cough, and nasal congestion. He was diagnosed with pneumonia, given one dose of intramuscular ceftriaxone, and given a five-day course of oral cefdinir with subsequent improvement of his symptoms. Three days prior to admission, the patient’s fever, rhinorrhea, and cough worsened. On the day prior to admission, the patient developed projectile non-bloody, bilious emesis. In the ER, the patient appeared distressed, had a fever (38.0℃), heart rate of 168 beats per minute, and a respiratory rate of 30 breaths per minute.

**Figure 1:**
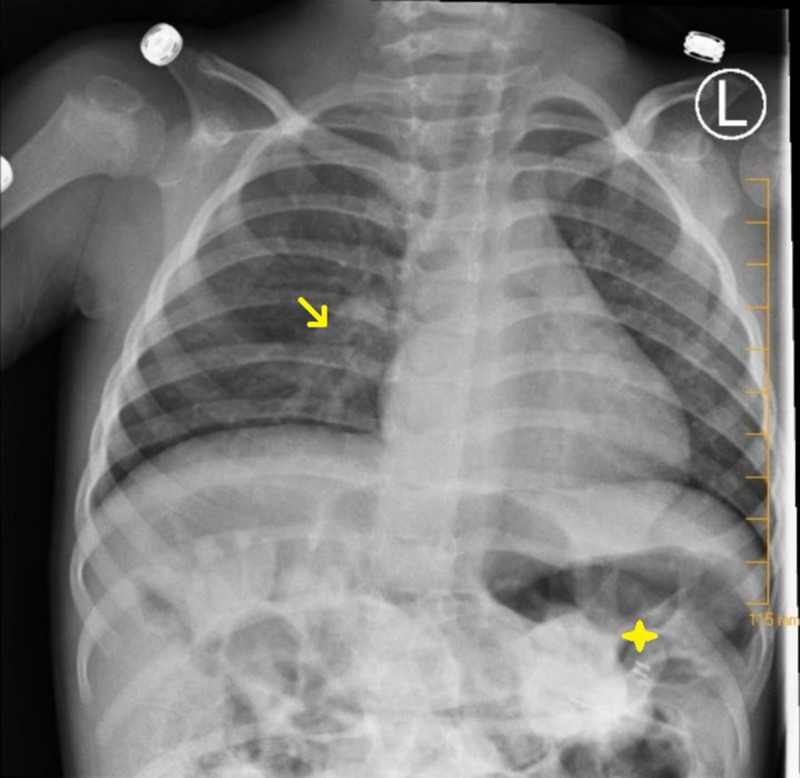
X-ray chest, PA view: mild increase in perihilar bronchial wall thickening and interstitial opacities suggestive of viral chest infection (arrow). No focal consolidation to suggest bacterial pneumonia. No pleural effusion or pneumothorax. Percutaneous gastrostomy tube visible (star). L, Left; PA, posteroanterior

**Figure 2:**
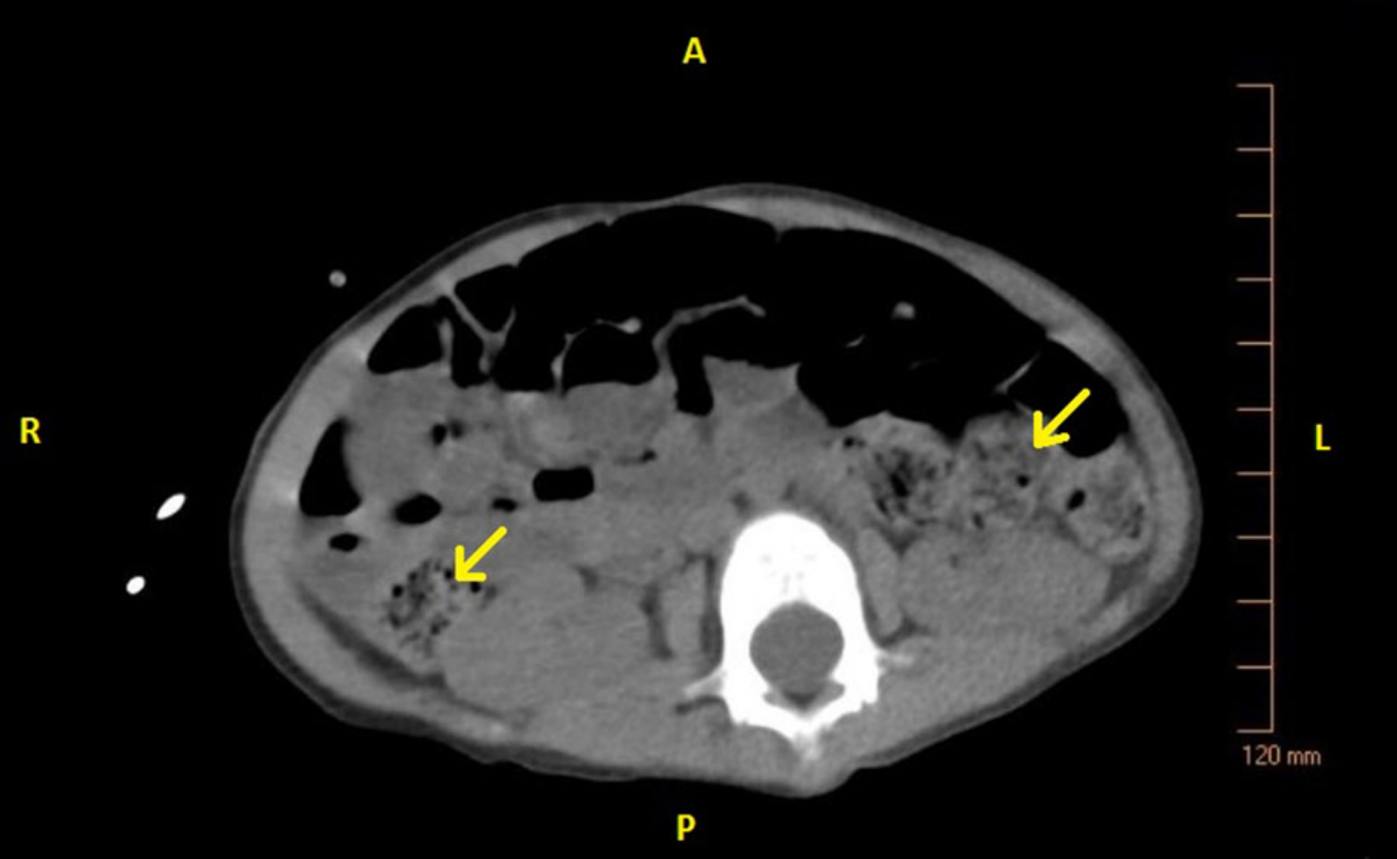
CT abdomen non-contrast, transverse view: large amount of stool and gas throughout the colon (arrows). A, Anterior; P, Posterior; L, Left; R, Right; CT, computed tomography

**Figure 3:**
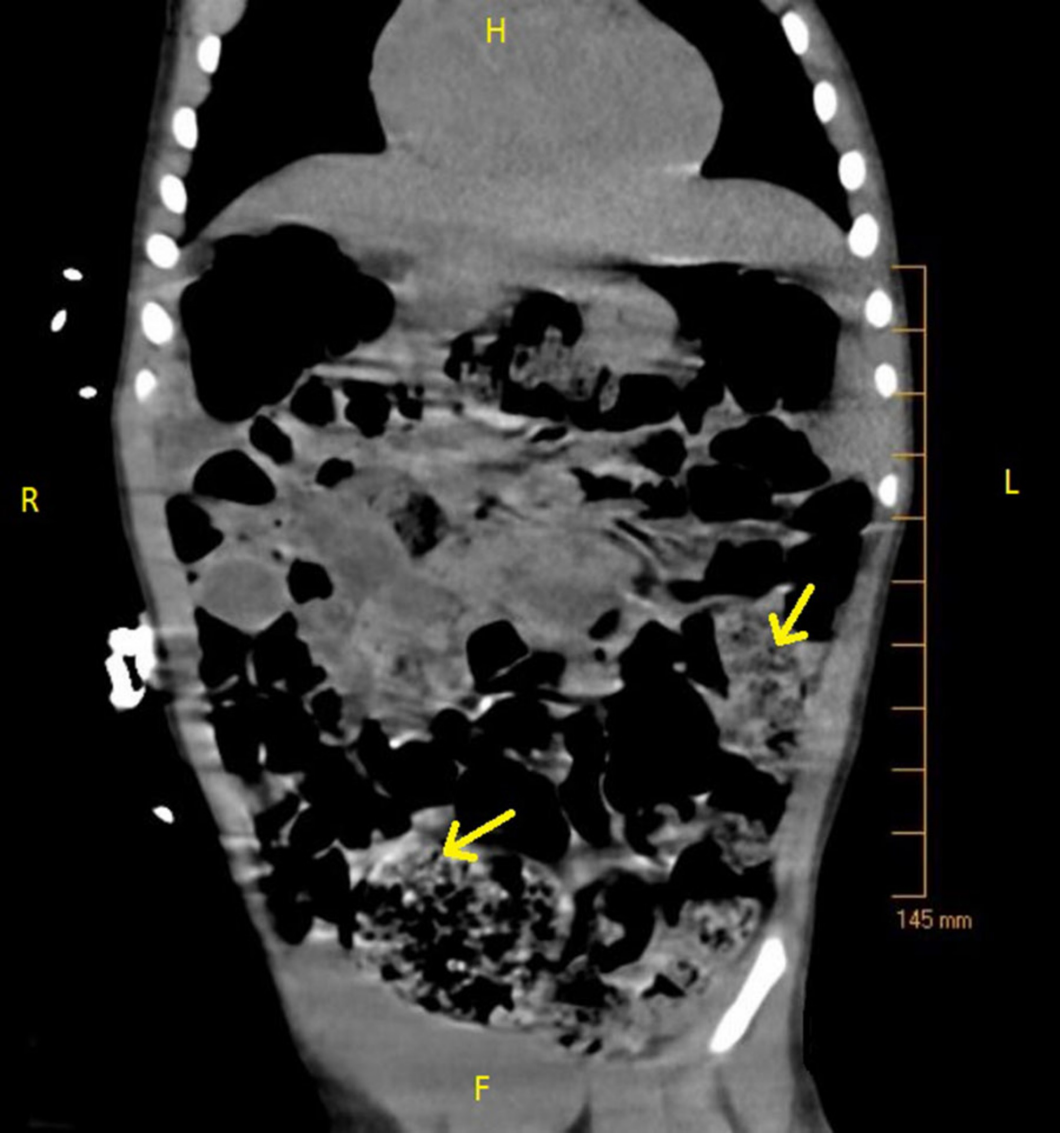
CT abdomen non-contrast, coronal view: large amount of stool and gas throughout the colon (arrows). H, Head; F, Foot; L, Left; R, Right; CT, computed tomography

On admission, the patient was placed on oxygen via nasal cannula and given acetaminophen, ketamine, and fentanyl for pain control. Following medication, the physical exam yielded a happy child in a non-toxic state. His abdomen was soft, non-tender, with normal bowel sounds. The liver edge was palpable, and his gastrostomy tube (G-tube) feeding site was free of infection. A chest X-ray was consistent with viral pneumonia as mild perihilar bronchial wall thickening and interstitial opacities were visualized (Figure 1). An abdominal computerized tomography (CT) scan showed copious amounts of stool in the large intestine without evidence of intussusception, appendicitis, colitis, or a volvulus (Figures 2-3). The patient’s parents stated his last bowel movement was one day prior and was semi-soft and normal. They also stated that he typically stooled a small amount, several times a day. A viral panel from a nasopharyngeal swab was ordered, which was positive for enterovirus. All other labs were within normal limits. The patient was diagnosed with viral gastroenteritis and pneumonia.

Constipation remained an issue despite the diagnosis of viral gastroenteritis. The patient was given polyethylene glycol for treatment of his constipation. After a period of no bowel movements, the dose was increased. Subsequently, the patient had two medium bowel movements and one smaller movement during the night.

The patient received intravenous (IV) normal saline at 30 mL/hr for presumed dehydration along with IV ceftriaxone at admission. The patient pulled out the IV during his hospital stay, and IV antibiotic access was not further pursued or continued due to viral infectious etiology. The patient took polyethylene glycol during his stay for his constipation. The patient was given nutrition via his G-tube.

The patient was discharged after improving on oral polyethylene glycol with instructions on oral rehydration and home-based monitoring. 

## Discussion

NALCN channelopathies have been discovered only in recent years and much needs be learned about the clinical implications in persons exhibiting genetic variations of the channel. As a known phenotype of a NALCN mutation, the UNC80 gene mutation affects neuron excitability leading to various hypotonic presentations in affected patients [3]. In our patient, this hypotonia caused a hospital stay for a routine viral infection and an overall complicated clinical course.

Firstly, dysfunctional muscle coordination causes severe constipation and feeding difficulties, often requiring the use of a G-tube to meet nutritional requirements [6-7]. Severe constipation and G-tube insertion have been previously documented in patients with the UNC80 mutation. More research is needed into the pathophysiology of these gastrointestinal disturbances and their implications. The patient had demonstrated dysfunctional bowel habits prior to admission with reports of small, hard movements daily that also were likely due to his severe hypotonia and intestinal muscle coordination dysfunction. After treatment in the hospital, he still displayed small bowel movements which were only semi-soft in consistency.

In addition, the patient required extensive pain medication for constipation to relieve severe discomfort demonstrated by his repeated pelvic thrusting. Treatment of his discomfort with opiates would have only further complicated his constipation. There have been no published reports of decreased gastrointestinal motility increasing susceptibility to viral gastrointestinal syndrome inpatients with UNC80 gene mutations. Our patient had difficulty recovering from his gastrointestinal infection needing a hospital stay as his inability to generate diarrhea hindered his expulsion of the viral load. His impaired respiratory clearance also contributed to the development of pneumonia in the setting of chronic lung disease, requiring various treatment modalities.

## Conclusions

The UNC80 mutation is a newly discovered mutation and much is left to be studied on the subject. More reports and studies are needed to fully understand the clinical implications of the mutation and its phenotypes and so medical professionals can improve treatments and better predict outcomes for patients affected by this disabling genetic anomaly.
